# Combining Cardiorespiratory Signals and Video-Based Actigraphy for Classifying Preterm Infant Sleep States

**DOI:** 10.3390/children10111792

**Published:** 2023-11-07

**Authors:** Dandan Zhang, Zheng Peng, Carola Van Pul, Sebastiaan Overeem, Wei Chen, Jeroen Dudink, Peter Andriessen, Ronald M. Aarts, Xi Long

**Affiliations:** 1 Department of Electrical Engineering, Eindhoven University of Technology, 5612 AP Eindhoven, The Netherlands; d.zhang3@tue.nl (D.Z.); z.peng@tue.nl (Z.P.); c.v.pul@tue.nl (C.V.P.); s.overeem@tue.nl (S.O.); r.m.aarts@tue.nl (R.M.A.); 2 Department of Personal and Preventive Care, Philips Research, 5556 AE Eindhoven, The Netherlands; 3 Department of Clinical Physics, Máxima Medical Center, 5504 DB Veldhoven, The Netherlands; 4 Sleep Medicine Center, Kempenhaeghe, 5591 VE Heeze, The Netherlands; 5 The Center for Intelligent Medical Electronics, School of Information Science and Technology, Fudan University, Shanghai 200433, China; w_chen@fudan.edu.cn; 6 Department of Neonatology, University Medical Center Utrecht, Wilhelmina Children’s Hospital, 3584 EA Utrecht, The Netherlands; j.dudink@umcutrecht.nl; 7 Department of Neonatology, Máxima Medical Center, 5504 DB Veldhoven, The Netherlands; p.andriessen@mmc.nl

**Keywords:** sleep-state classification, preterm infant, cardiorespiratory signal, video-based actigraphy

## Abstract

The classification of sleep state in preterm infants, particularly in distinguishing between active sleep (AS) and quiet sleep (QS), has been investigated using cardiorespiratory information such as electrocardiography (ECG) and respiratory signals. However, accurately differentiating between AS and wake remains challenging; therefore, there is a pressing need to include additional information to further enhance the classification performance. To address the challenge, this study explores the effectiveness of incorporating video-based actigraphy analysis alongside cardiorespiratory signals for classifying the sleep states of preterm infants. The study enrolled eight preterm infants, and a total of 91 features were extracted from ECG, respiratory signals, and video-based actigraphy. By employing an extremely randomized trees (ET) algorithm and leave-one-subject-out cross-validation, a kappa score of 0.33 was achieved for the classification of AS, QS, and wake using cardiorespiratory features only. The kappa score significantly improved to 0.39 when incorporating eight video-based actigraphy features. Furthermore, the classification performance of AS and wake also improved, showing a kappa score increase of 0.21. These suggest that combining video-based actigraphy with cardiorespiratory signals can potentially enhance the performance of sleep-state classification in preterm infants. In addition, we highlighted the distinct strengths and limitations of video-based actigraphy and cardiorespiratory data in classifying specific sleep states.

## 1. Introduction

Sleep plays a vital role for preterm infants. It has a significant impact on their early neurosensory development [1,2,3,4,5,6,7] and physiological processes [8,9]. During sleep, the brain engages in specific processes, including synaptic pruning and neural network refinement. These processes are indispensable for the formation of memory and long-term memory circuits and for sustaining brain plasticity throughout their lifetime [10,11]. Sleep has profound effects on the developmental physiology of infants as well. As the preterm infant develops towards full term, marked changes in the cardiorespiratory system can be observed between different sleep states [12,13,14] and between sleep and wake [15].

Based on observation of preterm infants’ behaviors, wake and sleep are distinguished, and sleep can be further divided into active sleep (AS), quiet sleep (QS) [16]. In the present paper, sleep states in general mean AS, QS, and wake unless otherwise mentioned. Many studies have shown that fetuses and newborns exhibit spontaneously discrete and cyclic patterns of AS and QS [17]. The physiological difference between AS and QS is significant, making it easier to distinguish between them [18].

The gold standard for monitoring sleep is using polysomnography (PSG), which uses several adhesive electrodes that are attached to the infant’s skin to monitor brain activity, eye movements, muscle tone, and other physiological parameters [19,20,21]. However, PSG is not feasibly available for preterm infants due to their fragile health condition. The application of multiple obtrusive contacts on the body of preterm infants increases the risk of skin infection and will in fact disturb their sleep [22,23,24].

Due to the possibility of being measured using unobtrusive or contactless sensors, cardiorespiratory signals are intensively studied in assessing sleep states, in particular in detecting AS and QS [25]. Electrocardiography- (ECG-) derived heart rate (HR) and heart rate variability (HRV) display different characteristics in each sleep state. During QS, HR decreases, while in AS, it increases, accompanied by changes in HRV’s frequency and time domain characteristics [26,27]. As for respiration, the regularity of respiration rate (BR) and the depth of respiration changed over the sleep states, where the BR shows a more regular pattern in QS [28,29], and the tidal volume of respiration in AS is higher than in QS [30]. Additionally, we also considered cardiorespiratory interaction (CRI), which shows a more regular and stratified clustered pattern during QS in comparison to other states [31]. However, the distinction between AS and Wake is challenging due to certain similarities in their physiological and behavioral characteristics [32].

Video-based monitoring is a contactless method that overcomes the limitation of PSG on sleep research [33,34]. It works, for example, by capturing the subject’s body movement, which has been demonstrated to be highly correlated with the infant’s wake/sleep state. Extensive research into the sleep of adults and term infants has produced promising results by analyzing wrist-worn actigraphy [35]. Given the fact that actigraphy can provide a reasonable validity and reliable way for assessing the development of infants’ sleep, video-based actigraphy (i.e., actigraphy estimated from video) [36] may provide a more unobtrusive and effective way to assess infant sleep.

This study aimed to validate video-based actigraphy for sleep-state classification in preterm infants and assess the potential of combining video-based actigraphy with cardiorespiratory signals to enhance sleep classification performance in this population. To achieve this objective, an extremely randomized trees (ET) classifier was performed with leave-one-subject-out cross-validation on cardiorespiratory signals, video-based actigraphic data, and their combination in each part on infant sleep state assessment.

## 2. Materials and Methods

### 2.1. Data and Annotation

This study was approved by the medical ethical committee (Medisch Ethische Toetsingscommissie, METC) of the Máxima Medical Center (MMC) on 23 November 2011 and was conducted in the neonatal intensive care unit (NICU) at MMC in Veldhoven, The Netherlands, in 2012. The parents of the preterm infants signed the informed consent. Eight preterm infants with an average gestational age (GA) of 30.0 ± 2.7 weeks, an average postmenstrual age (PMA) of 32.5 ± 2.8 weeks, and an average birth weight of 1680.8 ± 634.3 g were involved in this study. The anthropometric characteristics of these preterm infants are summarized in Table 1.

Cardiorespiratory signals (ECG and respiratory signals) and videos were recorded simultaneously during the routine regular monitoring. The ECG signal was recorded at 500 Hz and the respiratory signal at 16 Hz by chest impedance electrodes, collected using the regular bedside patient monitor (Philips Monitor Intellivue MX800, Böblingen, Germany). To reduce variability between subjects or recordings, ECG and respiratory signals were normalized (Z-score normalization) to obtain a mean of zero and a standard deviation of one for each recording before feature extraction. Moreover, CRI was constructed based on the timing of the R peak of each heartbeat in the ECG signal and the amplitude of the respiratory signal at that corresponding time stamp [31]. For video recordings, the video resolution and frame rate (measured by the IDS uEye Monochrome camera, Obersulm, Germany) were 736 × 480 pixels and 8 Hz, respectively. The position of the camera was fixed and the entire body of preterm infants with a direction of “foot to head” was in the vision of the camera.

The recordings were annotated into five states based on 30 s non-overlap windows (epochs): AS, QS, Wake, Caretaking, and Unknown (unable to annotate) by an experienced sleep expert based on the ruler of sleep annotation system [16]. Because in our data set, preterm infants were always awake during caretaking periods, generating very similar signal structures, the labels caretaking and wake were merged into the label caretaking + wake (CTW), representing wake in this work [30,37]. The unknown states were excluded from the analysis. This resulted in a total of 39.31 h of data used in this study with an average recording time of 4.91 ± 1.45 h (589 ± 174 epochs) for the 8 preterm infants and the resulting overall distribution of sleep states was AS = 72%, QS = 12% and CTW = 16%.

### 2.2. Feature Extraction

A total of 91 features were extracted from time, frequency, and non-linear domains from ECG, respiratory signal, CRI, and video data, resulting in 34 features extracted from cardiac signals, 41 features adapted from respiratory activity, 8 features from CRI, and 8 new features from video.

The cardiac features comprise existing HRV features within the specific frequency domain. The frequencies of preterm infants are categorized as very low frequency (VLF), low frequency (LF), and high frequency (HF). Additionally, the spectrum power in the two extended high frequencies, sHF and uHF, were also computed. To account for noise caused by the movement of capacitive electrodes, the Beats per epoch counts the R peaks, while the Line length and mean Line length, along with their standard deviation, were calculated over the length of the ECG time series signal. For capturing regulatory changes reflecting the autonomic nervous system response in preterm infants, the percentage of HR decelerations and the magnitude of HR deceleration were determined. The non-linear sample entropy, sample entropy area under the curve, and quadratic sample entropy were computed to depict the autonomic response in preterm infants. Additionally, the Lempel–Ziv complexity measure quantified the fluctuation of the ECG and its corresponding HRV signals, and more detail can be found in our previous work [37].

The previous work included revising the respiration features by extracting various parameters from the respiratory effort signal. These parameters were influenced by Boe et al. [38]. They included the variance, median, and standard deviation of the respiratory frequency, the logarithm of respiration frequency per minute, the relative change in frequency per second, and the breath-by-breath correlation (mean, standard deviation, and minimum and maximum). The features in the respiratory spectrum, such as respiratory frequency and power, were adapted from Redmond et al. [39]. This involved extracting the logarithm and normalized spectral power in VLF, LF, and HF bands, as well as the VLF-to-HF ratio and the LF-to-HF ratio. Additionally, the total power of the respiration frequency spectrum, the dominant frequency spectrum of respiration, and the logarithm of the spectrum of the dominant frequency power were calculated. Estimating respiratory amplitude features, as suggested by Long et al. [40], involved determining the standardized median of the amplitude of peaks and troughs, the approximate entropy of respiratory peaks and troughs, the median peak-to-trough difference, and the median and standard deviation of the logarithm of the respiratory peak-to-trough ratio. Other features extracted included the median volume and flow rate for a complete breath cycle, inhalation, and exhalation, as well as the ratio between the medians of respiratory time and respiratory rate during the inhalation-to-exhalation flow rate ratio. The average, standard deviation, and delta of the amplitude ratio were also calculated. Furthermore, the measurement of respiratory regularity using sample entropy was revised based on the work of Richman et al. [41].

Several parameters of the visibility graph (VG) network constructed from the CRI time series showed significant differences across preterm infant sleep states [31]. As a result, we gathered 8 features from the CRI signal. We calculated the mean and standard deviation of the degree of both the VG-based CRI network and the differencing VG-based network (DVG). The assortative coefficient (AC) was used to measure the assortative mixing based on degree, representing the skewness of network node connections. We also considered the clustering coefficient (CC) to measure the density of local clusters in the network, resulting in the mean and standard deviation of CC values calculated per epoch. To quantify the regularity of the CRI network, we computed the sample entropy (SE) of network degrees. Furthermore, we extracted features from the degree distribution to examine the statistical properties of the CRI network.

Movement, captured from video, can be used as an indicator for certain sleep states. Motion values were estimated from video frames based on the 3-dimensional recursive search (3DRS) algorithm [42].

Eight motion features were included. Mean motion count (Motion_mean_) refers to the average motion amplitude within one 30-s epoch. The greater the amplitude, the greater the movement during sleep. Standard deviation motion count (Motion_sd_) describes the deviation of motion amplitude within one epoch, which measures the stability of movement during sleep. Motion_sum_ is the sum of motion amplitude within one epoch. Motion_count_ is the number of motion amplitudes larger than zero within one epoch, and an exponentially weighted moving average is applied to smooth the activity count for each epoch. Moreover, to further quantify the distribution of the value of motion count, percentiles *P*_25_, *P*_50_, *P*_75_, and *P*_95_ were extracted.

The overview of all the features is in Tables S1–S4 in the Supplementary Materials.

### 2.3. Feature Preprocessing

The features were preprocessed to enhance their applicability before being fed into the training model. There is a highly imbalanced distribution of sleep states; however, machine-learning algorithms are typically designed for balanced datasets, and an imbalanced dataset can lead to a bias towards the majority class, resulting in poor performance in the minority class [43]. Therefore, it is crucial to address the imbalanced dataset prior to training the model to improve its reliability. The synthetic minority oversampling technique (SMOTE) is a widely used technique that creates synthetic instances in the minority class, improving class balance and mitigating the problems of overfitting and poor generalization [43]. In this study, we applied SMOTE to address the issue of imbalanced datasets. To avoid problems with overfitting and poor generalization, we split the data into training and test sets before applying oversampling techniques. This ensures that the same observations are not present in both sets, preventing the model from simply memorizing specific data points.

### 2.4. Classification Algorithms

The ET [44] classifier is a tree-based algorithm and was created based on the idea of ensemble learning. It works by constructing a set of decision trees during training and giving the final decision by aggregating the predication from these trees. During the tree-building process, ET constructs trees with different subsets of features and assigns importance scores to features. By utilizing these scores, ET can effectively identify and select the most relevant features, enhancing model performance by reducing noise and dimensionality in the dataset. This classifier is known for its robustness, low variance, and low bias, and is a fast-to-train algorithm with good predictive performance. ET is available in the Python Scikit-learn library [45].

Tree-based classifiers, in particular ET, have shown good performance in preterm infant sleep-state classification [26], which was therefore chosen in this work. Classification was performed on three different feature combinations (i.e., feature sets):

1. The motion features from video-based actigraphy (Motion).
2. The ECG, respiration, and CRI features (ECG-Resp-CRI).
3. The ECG, respiration, CRI, and motion features (ECG-Resp-CRI-Motion).

Feature importance was determined by utilizing the intrinsic characteristics (Gini importance) of ET. Additionally, we classify different paired sleep states (AS vs. QS, QS vs. CTW, AS vs. CTW, and Sleep vs. CTW), aiming to gain deeper insights into the features and the performance in distinguishing between each two sleep states.

### 2.5. Model Evaluation

Cross-validation is primarily used to evaluate the effectiveness of a machine-learning model on unseen data when the dataset is relatively small. The choice of the cross-validation method depends on several factors, including the sample size, the experimental design, and the research problem. In this study, leave-one-subject-out cross-validation was used due to the limited sample size and to ensure complete separation between the training and test data, ensuring that data were finally tested on a completely new patient, as also would be its use in later clinical practice.

**Figure 1:**
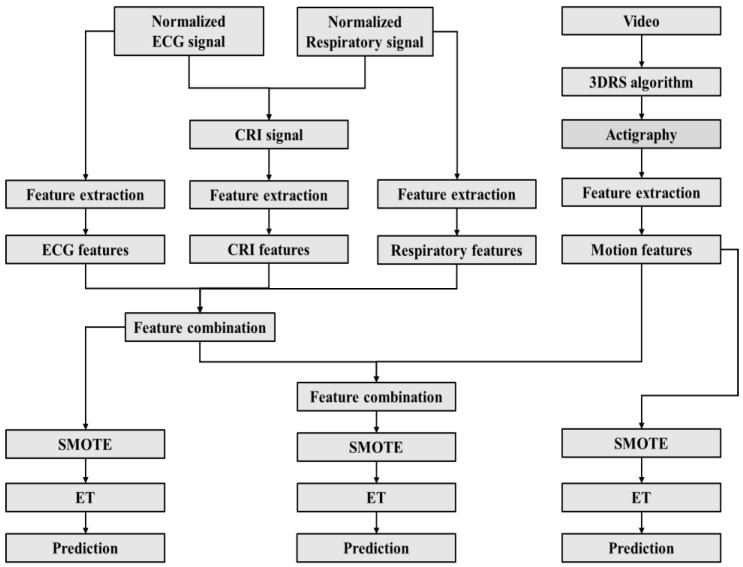
Flowchart for automated sleep classification based on different feature sets. CRI: Cardiorespiratory interaction; 3DRS: 3D recursive search (3DRS) motion estimation algorithm. SMOTE: Synthetic minority oversampling technique. ET: Extremely randomized trees.

The sleep classification performance was evaluated using commonly used metrics, including accuracy, precision, sensitivity, specificity, and the area under the receiver-operating-characteristic (ROC) curve (AUC). Cohen’s kappa score was also used due to the unbalanced distribution of classes [46] and was selected as the standard to determine the base and meta classifiers. Furthermore, confusion matrices were used to provide a comprehensive visualization of the classification performance. Figure 1 gives an overview of the flowchart for automated sleep classification for this study.

## 3. Results

Table 2 compares the sleep-state classification performance in preterm infants using different feature sets and ET. A low kappa score (0.26 ± 0.12) was achieved when using only motion features derived from video-based actigraphy, clearly lower than that (kappa = 0.33 ± 0.11) using cardiorespiratory signals. The combination of motion and cardiorespiratory features led to an improved kappa score of 0.39 ± 0.11.

**Figure 2:**
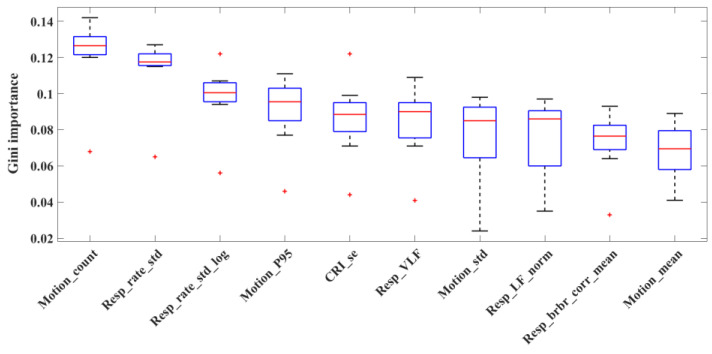
Box plots of the top 10 features in sleep-state classification based on Gini importance across subjects. +: The outliers. Feature names and their description can be found in the Supplementary Material.

To understand the importance of features in classifying sleep states, Figure 2 shows the top 10 features ranked based on their Gini importance score when using all features, including five respiratory features, one CRI feature, and four motion features.

The classification performance of the ET model on the three feature sets is shown in Table 3. Among the feature sets, ECG-Resp-CRI exhibited the highest sensitivity for AS and QS, as well as precision for CTW. Interestingly, a high sensitivity (0.60) for CTW was achieved using motion features solely, indicating their capability to detect wake (and caretaking) from sleep, which eventually contributed to the classification model when combining them with cardiorespiratory features.

**Figure 3:**
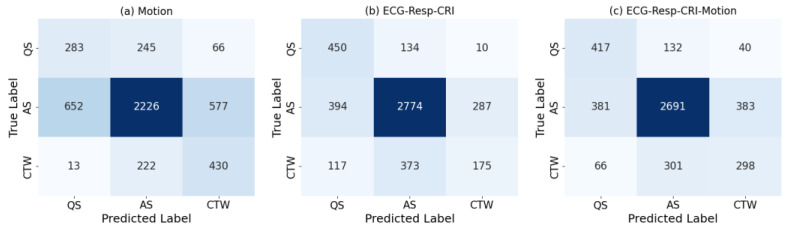
Confusion matrices in preterm infant sleep-state classification (AS, QS, and CTW) using feature set: (**a**) Motion; (**b**) ECG-Resp-CRI; (**c**) ECG-Resp-CRI-Motion. The aggregated results of all epochs are presented.

Figure 3 displays the confusion matrices of sleep-state classification with different feature sets. The largest correctly predicted numbers for AS and QS were achieved when using ECG-Resp-CRI features. Moreover, a notable augmentation in CTW detection was observed when incorporating motion features during sleep, increasing the correctly predicted number of CTW from 175 to 298 epochs compared to using ECG-Resp-CRI features alone.

Table 4 presents the binary classification results obtained from ET. Notably, the ECG-Resp-CRI feature set demonstrated a good performance in distinguishing between AS and QS, as well as QS and CTW. However, challenges arose when attempting to differentiate between AS and CTW, as well as Sleep and CTW. We can also see from the table that the motion features clearly outperformed the cardiorespiratory features (ECG-Resp-CRI) in discriminating between CTW and AS, as well as between CTW and Sleep. In general, using all features, the model performed the best, showing a similar kappa value in classifying AS and QS (0.48 ± 0.17), but showed a much better performance in classifying CTW and sleep (including AS and QS) in comparison with only cardiorespiratory features.

**Figure 4:**
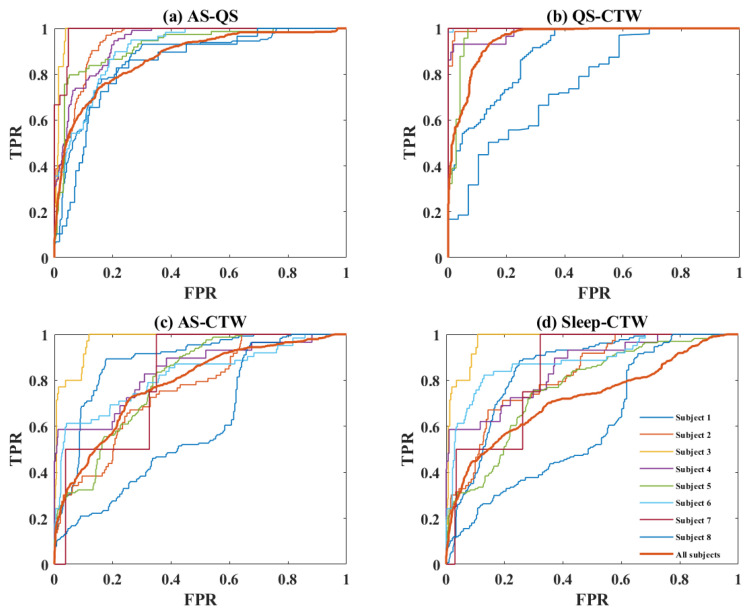
ROC curves for different binary classifications using all cardiorespiratory and motion features for each subject and for all subjects (epochs pooled over subjects). FPR: False positive rate; TPR: True positive rate.

To acquire more comprehensive insights into the performance of ET in multi-classification, we analyzed the ROC for each pairwise binary classification based on all the feature sets. The results are shown in Figure 4. It is obvious that the discrimination between QS and CTW exhibited better performance for most patients (AUC = 0.95 ± 0.09, kappa = 0.80 ± 0.25) compared to the others, especially between AS and CTW (AUC = 0.81 ± 0.11, kappa = 0.31 ± 0.17), which was difficult to separate. Nevertheless, relatively large variability between subjects in terms of classification performance can be observed in the figure.

## 4. Discussion

The study demonstrates the potential advantages of incorporating motion features captured from video into the regularly used cardiorespiratory features for the classification of the sleep states of preterm infants. To achieve this objective, the classification of sleep states using different combinations of features was implemented based on an ET algorithm.

It is particularly important to note that several motion features are highly ranked, indicating their important contribution in boosting the classification intuitively due to their capability to detect wake and caretaking from sleep. In a study that was conducted by Ho et al. [47], which investigated the differences in respiration patterns between extremely preterm infants (GA < 28 wk) and moderate preterm infants (32 ≤ GA < 35 wk) during sleep, it was observed that extremely preterm infants exhibited reduced respiration variability compared to moderate preterm infants. This observation aligns with the feature importance analysis that highlighted the significance of respiration variability. However, due to the limitation of the small sample size in the current study, future studies could investigate more preterm infants with a broader gestational age range to gain further insights into the impact of respiration on sleep states. Notably, the ECG features did not reach the top 10 when only looking at general performance, though while performing detailed analysis on different discriminating tasks in this study, it became clear that they have a role in discriminating between active and quiet sleep, as also observed in other papers [37]. These findings align with the research conducted by Long et al. [48], which established the reliability of respiratory effort and actigraphy as physiological signal modalities for sleep and wake classification in adults. Similarly, Karlen et al. [49] enhanced (wrist-worn) actigraphy-based sleep–wake classification using the respiratory signal. The importance of including motion features resulted in enhancing the model’s sensitivity and precision in identifying AS and CTW, as well as Sleep and CTW. The significance of movement in sleep assessment arises from two main reasons. Firstly, movement offers insights into respiratory patterns, including irregular breathing, which is a frequent concern in preterm infants. Secondly, assessing movement provides a straightforward method for observing sleep–wake patterns. Our study further confirms this efficacy by showing similar outcomes in the classification of Sleep and CTW. This assertion is supported by the research of Ülgen et al. [50], which established actigraphy as a widely used method for measuring sleep–wake patterns. Additionally, we refer to the work of Schmidt et al. [51] and Hyde et al. [52], who concluded that actigraphy is a valid approach for monitoring sleep in infants. The integration of motion features has been proven to be a valuable approach to sleep-state classification. These findings not only support the usefulness of motion features in sleep-state classification [53] but also demonstrate the feasibility and reliability of the motion detection algorithm utilized in our study, which was based on video analysis.

In addition to the effectiveness of motion features, Table 3 also indicates that features reflecting cardiorespiratory information play a crucial role in classifying AS and QS. These findings align with the results in Table 4 and Figure 3b, further confirming the consistent and valuable contribution of cardiorespiratory features in accurately differentiating between these specific sleep states (AS and QS, as well as CTW), where motion features fail to make this distinction. The finding aligns with prior research by Werth et al. [20] and Bourel-Ponchel et al. [54]. Their work demonstrated that AS and QS states exhibit dynamic and dominant changes during cardiorespiratory activity. However, differentiating between Sleep and CTW, in particular between AS and CTW, is challenging using only cardiorespiratory information, which warrants further investigation.

The combination of information from these two feature sets (ECG-Resp-CRI and Motion) enhances the overall effectiveness of the model in accurately classifying sleep states for preterm infants. We observed that there was a high variance in the kappa score between subjects, ranging from 0.14 to 0.51. This could imply that the model was dependent on the subjects used for training, yet it provided us with an idea about how the model might perform in worst-case and best-case scenarios when applied to new data. The limitation of the limited sample size should be addressed in future studies.

To further understand the process of sleep-state classification, we analyzed the ROC for each paired binary classification and observed that most subjects exhibited a better separation, except for subject 7 and subject 8. The ROC curves reflect the challenges in distinguishing between AS and CTW, as well as Sleep and CTW, for subject 7. Similarly, it highlights the difficulty in distinguishing between AS and QS, as well as Sleep and CTW, for subject 8. This result is consistent with our speculation that the model might be dependent on some specific subjects. For subject 7, the reason could be that there is an extremely imbalanced state proportion of CTW and AS, where there are only 5 epochs of CTW and 212 epochs of AS. It is not easy for a classification model that is designed for a balanced class to classify a skewed portion of classes. For subject 8, the bad results were likely due to the poor quality of raw signals, and this speculation was validated when we checked the video data where the illumination seemed insufficient when filming the baby.

This study has several limitations. First, the sample size is limited as mentioned before, with only eight infants included. Second, the study was hindered by a relatively low camera resolution and a dark environment, occasionally with insufficient illumination. Additionally, disturbances such as flashing lights from the patient monitor and shadows from caregivers might cause strong interference in videos. In the future, expanding the sample size to include more subjects and using a higher-resolution camera (or an infrared camera) is anticipated. Importantly, this study’s potential application lies in the integration of video-based respiration, pulse rate, and actigraphy, offering a non-intrusive method for assessing sleep in preterm infants.

## 5. Conclusions

For the automated sleep classification of preterm infants, we employed an extremely random tree classifier and achieved a fair agreement compared with an expert annotator. Moreover, our results suggest that cardiorespiratory features have limitations when it comes to detecting caretaking and wake from other sleep states and motion features have limitations in differentiating between active sleep and quiet sleep. Combining features from both video-based actigraphy and cardiorespiratory activity has been proven to be beneficial in compensating for each other’s shortcomings and contributes to an improvement in the classification performance. However, to achieve a more reliable and robust classification of sleep states in preterm infants, further investigation and improvement are necessary. Future work is encouraged to collect more data, improve the quality of video recordings, and consider integrating vital signals such as ECG, respiration, and movements all using video-based methods.

## Figures and Tables

**Table 1:** The anthropometric characteristics of the included preterm infants.

	Subject								Statistics	
	1	2	3	4	5	6	7	8	Mean	SD
GA (wk)	30.4	33.8	27.4	31.7	29.3	27.0	33.3	27.4	30.0	2.7
PMA (wk)	31.3	34.4	29.0	34.9	30.6	29.1	34.7	35.9	32.5	2.8
Weight (g)	1606	2410	1160	1845	1110	755	2080	2480	1680.8	634.3

GA: Gestational age; PMA: Postmenstrual age; SD: Standard deviation; wk: Week.

**Table 2:** Multi-classification results (kappa score) using ET based on different feature sets.

Subject	Kappa Score		
	Motion	ECG-Resp-CRI	ECG-Resp-CRI-Motion
1	0.48	0.41	0.33
2	0.15	0.39	0.41
3	0.30	0.21	0.45
4	0.26	0.49	0.51
5	0.27	0.43	0.35
6	0.13	0.29	0.44
7	0.41	0.27	0.46
8	0.12	0.14	0.14
Mean	0.26	0.33	0.39
SD	0.12	0.11	0.11

ET: Extremely randomized trees.

**Table 3:** Performance comparison using different feature sets in classifying preterm infant sleep states (AS, QS, and CTW) using ET. Results are presented in mean ± SD. The best performance of each metric is marked as bold.

Performance Metric	Feature Set		
	Motion	ECG-Resp-CRI	ECG-Resp-CRI-Motion
Accuracy	0.64 ± 0.12	0.70 ± 0.13	0.72 ± 0.12
Kappa	0.26 ± 0.12	0.33 ± 0.11	**0.39 ± 0.11**
Sensitivity AS	0.64 ± 0.18	**0.77 ± 0.15**	0.74 ± 0.20
Precision AS	0.82 ± 0.12	0.85 ± 0.10	**0.87 ± 0.11**
Sensitivity QS	0.46 ± 0.30	**0.74 ± 0.24**	0.69 ± 0.30
Precision QS	0.35 ± 0.28	0.51 ± 0.16	**0.53 ± 0.18**
Sensitivity CTW	**0.60 ± 0.32**	0.29 ± 0.28	0.51 ± 0.15
Precision CTW	0.38 ± 0.21	**0.51 ± 0.41**	0.40 ± 0.16

**Table 4:** Binary classification results displaying the kappa value for each subject per classification task. Sleep refers to AS + QS.

Feature Set	Sleep States	Subject								Statistic	
		1	2	3	4	5	6	7	8	Mean	SD
Motion	AS vs. QS	0.32	0.29	0.01	0.30	0.37	0.07	0.34	−0.02	0.21	0.15
	QS vs. CTW	0.63	0.60	0.53	0.67	0.47	0.75	0.63	0.21	0.56	0.15
	AS vs. CTW	0.63	0.15	0.49	0.36	0.23	0.59	−0.03	0.13	0.32	0.22
	Sleep vs. CTW	0.57	0.20	0.47	0.30	0.30	0.59	−0.03	0.14	0.32	0.20
ECG-Resp-CRI	AS vs. QS	0.31	0.57	0.47	0.54	0.56	0.42	0.76	0.38	0.50	0.13
	QS vs. CTW	0.80	0.62	1.00	0.91	0.89	0.30	1.00	0.04	0.69	0.33
	AS vs. CTW	0.00	0.00	0.00	0.27	0.36	0.18	0.01	0.00	0.10	0.04
	Sleep vs. CTW	0.01	0.00	0.04	0.24	0.31	0.00	0.00	0.00	0.07	0.12
ECG-Resp-CRI-Motion	AS vs. QS	0.15	0.57	0.43	0.56	0.59	0.41	0.76	0.37	0.48	0.17
	QS vs. CTW	0.59	0.94	1.00	0.88	0.94	0.87	1.00	0.21	0.80	0.25
	AS vs. CTW	0.34	0.20	0.46	0.50	0.28	0.56	0.07	0.09	0.31	0.17
	Sleep vs. CTW	0.29	0.39	0.50	0.52	0.34	0.55	0.06	0.10	0.34	0.17

## Data Availability

Due to privacy regulations, data are not available. Data were obtained from the Máxima Medical Center in Veldhoven, The Netherlands.

## Supplementary Materials

The following supporting information can be downloaded at: https://www.mdpi.com/article/10.3390/children10111792/s1, Table S1: ECG and HRV features; Table S2: Respiratory features; Table S3: Cardiorespiratory interaction features; Table S4: Motion features.
