# Supplementary material for: Combining Cardiorespiratory Signals and Video-Based Actigraphy for Classifying Preterm Infant Sleep States
Source: Children (Basel). 2023 Nov 7;10(11):1792. doi: 10.3390/children10111792 (PMC10670397; doi:10.3390/children10111792)
Supplement: Supplementary file 1 [file children-10-01792-s001.zip › children-2649880-supplementary.pdf]

Supplementary materials

Table S1 : ECG and HRV features

NR	Feature	Description
0	BpE	Beats per Epoch
1,2	LL, aLL	Line Length/mean Line Length
3-6	NNx	The number of pairs of successive RR intervals that differ by more than10, 20, 30 or 50 ms of a defined window length
7-10	pNNx	The proportion of NNx divided by the total number of R-R intervals of a defined window length
11	RMSSD	Root mean square of successive differences between adjacent R-R intervals of a defined window length
12	SDALL	Standard derivation of averaged line length
13	SDANN	Standard derivation of averaged NN intervals
14	SDLL	Standard derivation of line length
15	SDNN	The standard deviation of normal to normal R-R intervals of a defined window length
16	HF	The power of the high-frequency band between 0.15-0.4 Hz of a defined window size
17	HFnorm	HF power in normalized units HF/(Total Power-VLF) x 100
18	LF	The power of the low-frequency band between 0.04-0.15 Hz of a defined window size
19	LFnorm	LF power in normalized units LF/(Total Power-VLF) x 100
20	LF/HF	Ratio LF/HF
21	pHF_1	The power of the high-frequency band between 0.4-0.7 Hz
22	pHF_1norm	pHF_1 power in normalized units pHF_1/(Total Power-VLF) x 100
23	TotPow	Total power or variance of NN intervals of a defined window size
24	pHF_2	The power of the high-frequency band between 0.7-1.5 Hz
25	pHF_2norm	pHF_2 power in normalized units pHF_2/(Total Power-VLF) x 100
26	VLF	The power of the very-low-frequency band between 0.003-0.04 Hz of a defined window size.
27,28	SEN, QSE	Sample entropy / Quadratic sample entropy
29	SEAUC	Sample entropy area under the curve
30	pDEC	The percentage of HR decelerations
31	SDDec	Magnitude of HR deceleration
32,33	LZNN,LZECG	Lempel-Ziv complexity measure on HRV and ECG

Table S2 : Respiratory features

NR	Feature	Description
0	VLF	The logarithm of the spectral power in the very low frequency band between 0.01 and 0.3 Hz
1	LF	The logarithm of the spectral power in the low frequency band between 0.56 and 0.3 Hz
2	HF	The logarithm of the spectral power in the high frequency band between 0.56 and 1.1 Hz
3	VLF/HF	The ratio between VLF and HF spectral powers
4	LF/HF	The ratio between LF and HF spectral powers
5	VLFnorm	VLF power in normalized units of respiration, VLF/TotPow x 100
6	LFnorm	LF power in normalized units of respiration, LF/(TotPow-VLF) x 100
7	HFnorm	HF respiration power in normalized units, HF/(TotPow-VLF) x 100 of the respiration
8	TotPow	The total power of the respiration frequency spectrum
9	Resp_Var	Variance of respiration
10	Sampen	The sample entropy of respiration
11	Resp_freq	The spectrum the dominant frequency
12	Power of resp_freq	The logarithm of the spectrum the dominant frequency power
13	NR_breaths	The number of breaths within epoch
14	Rate	The median of frequencies
15	Rate_std	The standard deviation of frequencies
16	Rate_std log	The logarithm of breath per min
17	Rate delta	Relative change of frequency in 1/second
18	Duty cycle	Duty cycle of breathing
19	Amp ratio	Average amplitude ratio
20	Amp ratio std	The standard deviation of amplitude ratio
21	Amp ratio delta	The delta of amplitude ratio
22	Peak sdm	The amplitudes of respiratory peaks
23	Tough sdm	The amplitudes of respiratory troughs
24	Peak apen	The approximate entropy of the peak
25	Tough apen	The approximate entropy of the tough
26	Breath volume	The median respiratory volume (expressed by respiratory effort area) measured during breathing cycles
27	Breath fr	The median breath volume over time
28	Inhale volume	The median respiratory volume (expressed by respiratory effort area) measured during inhalation periods
29	Exhale volume	The median respiratory volume (expressed by respiratory effort area) measured during exhalation periods
30	Inhale fr	The median respiratory flow rate during inhalation periods
31	Exhale fr	The median respiratory flow rate during exhalation periods
32	PT distance	The ratio of the median peak-to-trough distance to peak-to-trough interquartile difference
33	PT ratio median	The logarithm of the median respiratory peak-to-trough ratio
34	PT ratio std	The logarithm of the standard deviation respiratory peak-to-trough ratio
35	In_ex_fr ratio	The ratio of the inhalation and the exhalation over time
36	In_ex_time ratio	The ratio of inhalation and the exhalation time ratio
37	Brbr corr mean	The mean of breath-by-breath correlation
38	Brbr corr max	The max of breath-by-breath correlation
39	Brbr corr min	The min of breath-by-breath correlation
40	Brbr corr std	The standard deviation of breath-by-breath correlation

Table S3. Cardiorespiratory interaction (CRI) features

NR	Feature	Description
0	CRI_Dm	Mean degree of VG network
1	CRI_Dsd	Standard degree of VG network.
2	CRI_Assr	The assortative coefficient that measures assortative mixing by degree and represents the skewness of node connections.
3	CRI_CCm	The average of clustering coefficient over all nodes.
4	CRI_CCstd	The standard deviation of clustering coefficient values
5	CRI_se	The sample entropy of the degree of the nodes
6	CRI_Ddm	Mean degree of DVG network
7	CRI_Ddsd	Standard degree of DVG network

Table S4 : Motion features

NR	Feature	Description
0	$Motion_{mean}$	The mean value of motion that was extracted based on ECG signal
1	$Motion_{std}$	The standard value of motion that was extracted based on ECG signal
2	$Motion_{sum}$	The sum of motion value that was extracted based on ECG signal
3	$Motion_{count}$	The number of motion value that were larger than zeros
4-7	$Motion_{Px}$	The number of motions that were larger than the x (x=25,50,75,95) percent of motion values that were extracted based on respiratory signal
